# Fabrication and Characterization of Al_2_O_3_-Siloxane Composite Thermal Pads for Thermal Interface Materials

**DOI:** 10.3390/ma17040914

**Published:** 2024-02-16

**Authors:** Seul-Ki Kim, Yeong-Jin Koo, Hyun Sik Kim, Jong-Keun Lee, Kyounghoon Jeong, Younki Lee, Eun Young Jung

**Affiliations:** 1 Semiconductor Materials Center, Korea Institute of Ceramic Engineering & and Technology, Jinju 52851, Republic of Korea; skkim@kicet.re.kr (S.-K.K.); kyj9118@kicet.re.kr (Y.-J.K.); 2 Department of Materials Engineering, Gyeongsang National University, Jinju 52828, Republic of Korea; 3 Analysis and Standards Center, Korea Institute of Ceramic Engineering and Technology, Jinju 52851, Republic of Korea; hyunkim@kicet.re.kr; 4 Daehan Ceramics Co., Ltd., Yeongam-gun 58452, Republic of Korea; jongkeun@dh-c.co.kr (J.-K.L.); samu21@dh-c.co.kr (K.J.); 5 Department of Materials Science and Engineering, Chonnam National University, Gwangju 61186, Republic of Korea; 6 Department of Materials Engineering and Convergence Technology, Gyeongsang National University, Jinju 52828, Republic of Korea; 7 The Institute of Electronic Technology, College of IT Engineering, Kyungpook National University, Daegu 41566, Republic of Korea

**Keywords:** thermal interface materials, flame fusion, Al_2_O_3_–siloxane composite thermal pad, spherical Al_2_O_3_ nanoparticle powder, thermal conductivity

## Abstract

In this study, Al_2_O_3_–siloxane composite thermal pads were fabricated using a tape–casting technique, and the thermal conductivity effect of the Al_2_O_3_ nanoparticle powder synthesized using a flame fusion process on siloxane composite thermal pads was investigated. Furthermore, various case studies were implemented, wherein the synthesized Al_2_O_3_ nanoparticle powder was subjected to different surface treatments, including dehydration, decarbonization, and silylation, to obtain Al_2_O_3_–siloxane composite thermal pads with high thermal conductivity. The experimental results confirmed that the thermal conductivity of the Al_2_O_3_–siloxane composite pads improved when fabricated using surface–treated Al_2_O_3_ nanoparticle powder synthesized with an optimally spheroidized crystal structure compared to that produced using non–treated Al_2_O_3_ nanoparticle powder. Therefore, this study provides guidelines for fabricating Al_2_O_3_–siloxane composite thermal pads with high thermal conductivity in the field of thermal interface materials.

## 1. Introduction

The rapid development of microelectronics technology has led to an increase in the integration and miniaturization of electronic components. However, there might be an increase in the heat generated by electronic devices during operation because of these innovations. Thermally conductive materials (TCMs) are used to ensure good heat transfer characteristics in electronic devices [1]. Among the different types of TCMs, thermally conductive adhesives, such as epoxy resins, are widely used in electronic devices because of their advantages, such as ease of processing, simple fabrication, low cost, and thermal stability [1]. The thermal conductivity of epoxy resins is very low, and it may be improved by filling the epoxy resin with ceramic, carbon, and metal particles [1]. However, incorporating metal particles deteriorates the electrical insulating and dielectric properties of these composites, thereby limiting their application to electronic packaging. Although carbon materials offer advantages such as high thermal conductivity and lightweight characteristics, their high cost and low electrical insulation properties limit their practical applications in industry. Ceramic particles provide excellent thermal conductivity and mechanical properties compared to carbon materials and metal particles, and, therefore, they have been extensively studied [1]. Ceramic materials are widely used in various fields of engineering, such as electronics, optics, metallurgy, and biomedicine [1,2]. Among them, Al_2_O_3_ is an attractive material for fabricating thermally conductive composite thermal pads because of its advantages, including high thermal conductivity, low cost, and stable chemical properties. In epoxy-resin composites, Al_2_O_3_ fillers increase the strength and elastic modulus of materials for product molding [1,2,3,4]. However, there are several challenges that are encountered when adding Al_2_O_3_ powder as a filler during the fabrication of thermal pads, such as molding failure and decreased rheological behavior of the Al_2_O_3_ paste. Therefore, numerous studies have investigated the material properties of the Al_2_O_3_ filling powder, including the particle size, shape, crystalline phase, polydispersity of particle size distribution, and elemental composition, which significantly influence the characteristics of Al_2_O_3_ pastes [5,6,7,8].

Given this context, this study investigated various surface treatments of Al_2_O_3_, including dehydration, decarbonization, and silylation. Furthermore, the structural properties were studied to overcome the Al_2_O_3_ nanoparticle agglomeration problem in trimodal distributions and improve the miscibility with siloxane to obtain highly conductive Al_2_O_3_–siloxane composite thermal pads. We focused on the surface modification of the spherical Al_2_O_3_ nanoparticle powder to fabricate Al_2_O_3_–siloxane composite thermal pads. The fabricated Al_2_O_3_-siloxane composite thermal pads were characterized using X–ray diffraction (XRD), field emission scanning electron microscopy (FE–SEM), Fourier transform infrared (FT–IR) spectroscopy, inductively coupled plasma-optical emission spectrometry (ICP–OES), nuclear magnetic resonance (NMR), and rheology, thermomechanical, and electrical properties.

## 2. Materials and Experimental Methods

### 2.1. Preparation of Al_2_O_3_ Nanoparticle Powder

**Figure 1:**
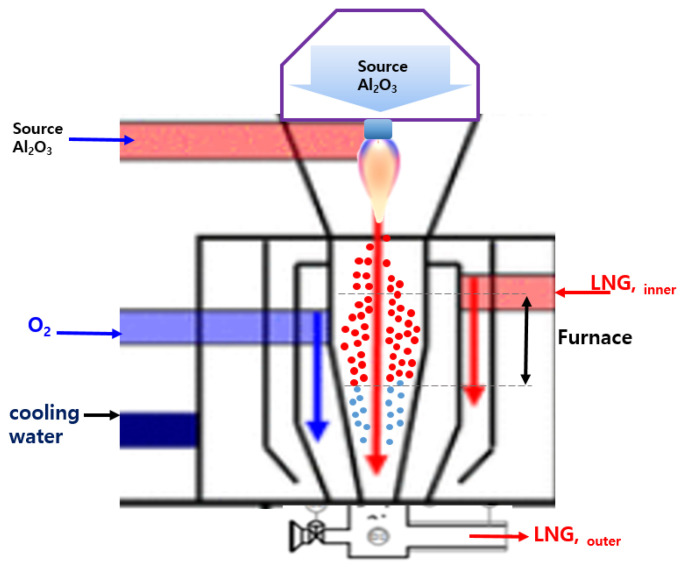
Experimental setup of the flame fusion process for spherical Al_2_O_3_ nanoparticle powders.

Figure 1 shows the experimental setup of the flame fusion process for synthesizing spherical Al_2_O_3_ nanoparticle powders. An Al_2_O_3_ nanoparticle powder with a particle size and purity of 300 nm and 99.9%, respectively, was used as the starting raw material.

Al_2_O_3_ powders were purified using deionized (DI) water and an acidic solution. The Al_2_O_3_ powders were spheroidized using three different liquefied natural gas (LNG) flow rates (60, 105, and 120 Nm^3^/h) to obtain the desired particle size. Propane and butane gases were primarily used. The Al_2_O_3_ raw powder was melted and spheroidized at 2500 to 3000 °C via the flame fusion process. The Al_2_O_3_ nanoparticle powders were synthesized by using a flame fusion process with three different LNG flow rates (60, 105, and 120 Nm^3^/h), and the obtained by–products had a high purity (>99.9%) and a mean diameter (D50) of 800 nm. The Al_2_O_3_ powders were continuously injected during the spheroidization process, and oxygen and LNG were flowed concurrently into the powder feeder. These combustion reactions occurred under the LNG flow as the following reactions [9]:

C_3_H_8_ + 5O_2_ → 3CO_2_ + 4H_2_O (1)

2C_4_H_10_ + 13O_2_ → 3CO_2_ + 10H_2_O (2)

The main parameters for spheroidization include three different LNG flow rates of 60, 105, and 120 Nm^3^/h at a fixed oxygen gas flow rate with a ratio of oxygen to LNG of 70:30. The Al_2_O_3_ powders in the reaction chamber treated under high–temperature conditions were quickly cooled after the synthesis process. In addition, when incomplete combustion occurs in the flame fusion spheroidization process using LNG, the generated CO_2_ or H_2_O is adsorbed on the surface of the Al_2_O_3_ nanoparticle powder and affects its physical properties. 

### 2.2. Surface Treatment of the Synthesized Al_2_O_3_ Nanoparticle Powders

In Case I (before treatment), since the obtained spherical Al_2_O_3_ nanoparticle powder contains a large amount of impurities adsorbed in the reaction chamber during synthesis, it is necessary to remove impurities from the surface of the Al_2_O_3_ nanoparticle powder in order to apply thermal pads with high thermal conductivity. Three different surface treatments were used for the synthesized Al_2_O_3_ powders, including dehydration (Case II), decarbonization (Case III), and silylation (Case IV). The surface of the Al_2_O_3_ powder was produced with a small amount of residual impurities, including acetaldehyde (CH_3_COH), hydrocarbons, and carbon-based components (CO_2_, CO), because of combustion reactions. Therefore, it was necessary to perform dehydration (Case II) and decarbonization (Case III) to remove these carbon compounds from the Al_2_O_3_ powder. For the dehydration reaction (Case II), the surface of the spherical Al_2_O_3_ powder was treated using a methanol solution. In this step (Case II), 50 wt% Al_2_O_3_ powder was added to the methanol solution and stirred for 30 min. After that, the treated Al_2_O_3_ powders were filtered by separating the processed powder and methanol via centrifugation. After filtration, the dehydrated Al_2_O_3_ powder was dried in a vacuum oven at 110°C under a N_2_ atmosphere for 24 h. In the case of decarbonization (Case III), the spherical Al_2_O_3_ powder was heated in a furnace at 750 °C for 1 h under ambient air conditions to remove the carbon compounds. 

For silylation (Case IV) with an aryl functional group (diphenyl–methoxy silane, molecular weight of 214.33 g/mol), the Al_2_O_3_ powder was added to a solution of methanol and 2 wt% silane and stirred for 30 min. The treated powders were filtered by separating the processed powder and methanol via centrifugation. After filtration, the dehydrated Al_2_O_3_ powder was dried in the oven at 110 °C for 24 h under ambient air conditions.

### 2.3. Fabrication of Al_2_O_3_-Siloxane Composite Thermal Pads Using the Synthesized Al_2_O_3_ Nanoparticle Powder

When using Al_2_O_3_ loading above 60 wt% of large particles in this experiment, the decrease in viscosity occurs in the high content of large particles by the Farris effect [10]. In general, the particle size of the Al_2_O_3_ powder will affect the thermal conductivity of the composite pads. This relationship between the thermal conductivity of the composite pad and its particle sizes is described as follows: the smaller the particle size, the better the thermal conductivity. This trend suggests that smaller particles can transfer heat more efficiently due to their high specific surface area [1]. However, the nanoparticles continuously thicken by aggregation in the liquid matrix, and then the next size-up particles could also be thicken this size distribution. For this reason, to prevent an increase in viscosity and create a composite thermal pad with high thermal conductivity, the synthesized spherical Al_2_O_3_ powder was applied in a trimodal system with three particle size distributions to fabricate Al_2_O_3_-siloxane composite thermal pads with high packing density.

**Figure 2:**
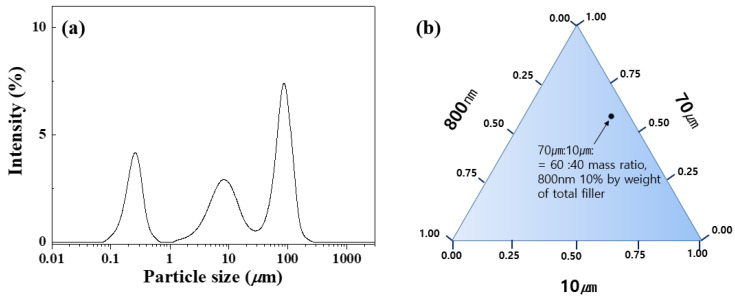
(**a**) Particle size distribution of Al_2_O_3_ powders and (**b**) percentage mixing ratio of the total filler for Al_2_O_3_–siloxane composite thermal pads. In this experiment, the mass ratio of 70 and 10 μm micro–sized Al_2_O_3_ powder was mixed with fixed conditions at a ratio of 6:4, respectively. Herein, 800 nm nanoparticle was used in 10% of the total filler.

Figure 2 shows the mixing ratio of the synthesized spherical Al_2_O_3_ powder. The trimodal mixture of the Al_2_O_3_ powder was blended using a solution blending process based on the mixing ratio, as indicated in Table 1. The blended Al_2_O_3_ powder mixtures were stirred for 24 h via wet mixing to achieve a homogeneous mixture. 

**Figure 3:**
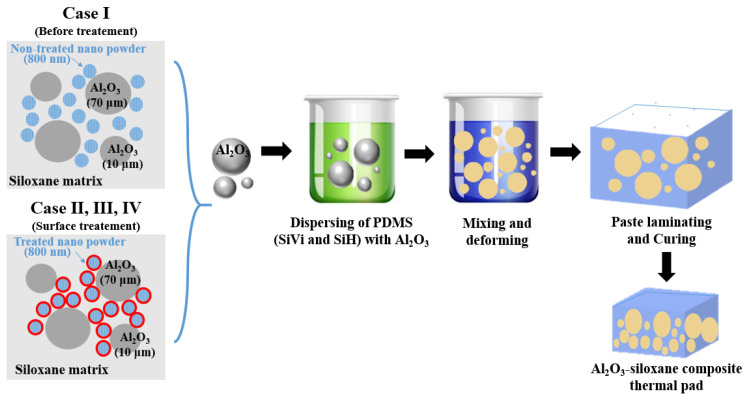
Scheme for preparing Al_2_O_3_–siloxane composite thermal pads with various surface–treated Al_2_O_3_ nanoparticle powders (Cases I, II, III, and IV) and a siloxane polymer.

Figure 3 shows the fabrication procedure of an Al_2_O_3_–siloxane composite thermal pad using a tape–casting technique with respect to various surface–treated nanoparticle powders (Cases I, II, III, and IV). For preparing Al_2_O_3_–siloxane composite pastes, the Al_2_O_3_ powder mixture was blended with vinyl–terminated polydimethylsiloxane (PDMS) combined with a silicone–hydride–terminated crosslinker (1:1 molar ratio with PDMS/silicone). The produced Al_2_O_3_–siloxane composite paste was homogeneously mixed for 1 h and maintained in a deformed state for 3 min to completely remove the air bubbles (AR–100, Thinky mixer, Tokyo, Japan). Finally, to fabricate the Al_2_O_3_–siloxane composite thermal pads, the Al_2_O_3_–siloxane composite paste was poured onto a Teflon substrate after mixing with a Pt catalyst (5 μL, 11.5 ppm). It was then laminated using a tape–casting technique and compressed at 25 °C under a pressure of 130 kg/m^2^ for 1 min. The laminated Al_2_O_3_–siloxane composite thermal pads were cured in an oven at 120° C for 8 h under ambient air conditions. The laminated Al_2_O_3_–siloxane composite thermal pads were removed from the Teflon substrate to obtain the Al_2_O_3_–siloxane composite thermal pads. Various Al_2_O_3_–siloxane composite thermal pad samples were systematically prepared using different volume fractions ranging from 60 to 79 vol% of Al_2_O_3_ powder to investigate the effects of the high contents of the Al_2_O_3_ powder.

### 2.4. Analysis and Characterization

#### 2.4.1. X-ray Diffraction 

The crystalline structure of the Al_2_O_3_ nanoparticle powder was measured using XRD (D/max–2500V/PC, Rigaku, Japan). To investigate the XRD peaks, XRD spectra were scanned over the θ–2θ range of 10° to 90° at 0.08 intervals using Cu Kα radiation (λ = 1.54 Å) at 40 mA and 100 kV. 

#### 2.4.2. Field Emission Scanning Electron Microscopy

The surface morphologies of the Al_2_O_3_ nanoparticle powder and Al_2_O_3_–siloxane composite thermal pads were evaluated using FE-SEM (JSM-7001F, JEOL, Tokyo, Japan) at a voltage and current of 20 kV and 0.8 nA, respectively. Before loading the samples into the chamber, they were made conductive by coating them with Pt. 

#### 2.4.3. Fourier Transform Infrared Spectroscopy

The main functional groups and crystalline phase of the Al_2_O_3_ nanoparticle powder were characterized using FT–IR spectroscopy (Vertex 70, Bruker, Billerica, MA, USA). The FT–IR spectrum was acquired using attenuated total reflection conditions at a wavenumber resolution interval of 0.6 cm^−1^ in the region from 650 to 4000 cm^−1^. 

#### 2.4.4. Inductively Coupled Plasma–Optical Emission Spectrometry

The elemental compositions and impurity quantifications of the Al_2_O_3_ powders were determined using ICP–OES (Optima 5300DV, Perkin Elmer, Waltham, MA, USA) for the cation analysis of the supernatant of the synthesized Al_2_O_3_ powders.

#### 2.4.5. Nuclear Magnetic Resonance

The Al_2_O_3_ nanoparticle powder was investigated using an ^27^Al solid–state NMR system (Avance 600 MHz, Bruker, Billerica, MA, USA) operated at a spinning speed of 22 kHz and 156.47 MHz for analyzing the aluminum (Al^+^) sites and structural transformations. All NMR spectra were calibrated using a 1M AlCl_3_ aqueous solution at 0 ppm as a reference. The peak deconvolution was quantified by spectrum simulation using quadruple coupling constants presented in the reference journal.

#### 2.4.6. Thermomechanical and Electrical Properties

The thermal resistance was measured at 50 °C in accordance with ASTM D5470 using a thermal resistance analyzer (T3Ster DynTIM, Siemens, München, Germany) to obtain the thermal conductivity of the Al_2_O_3_–siloxane composite thermal pads. For this measurement, the fabricated thermal pads were cut into 1 to 2 mm thick circles (diameter: 10 mm).

The thermal conductivity values of the Al_2_O_3_–siloxane composite thermal pads were calculated based on the changes in the thermal resistance at a fixed pressure of approximately 344.5 kPa. The thermal conductivity of the Al_2_O_3_–siloxane composite thermal pads was proportional to the slope of the curve obtained by plotting the measured thermal resistance values as a function of the distance between the measurement surfaces. These curves were obtained using Equations (3) and (4):

(3) $$k=\frac{∆L}{∆R_{th}}\times \frac{1}{A}=\frac{1}{m\times A}$$

(4) $$m=\frac{∆R_{th}}{∆L}$$

where k, ∆L, $∆R_{th}$, *A*, and *m* represent the calculated thermal conductivity of the material, width of the sample holder (i.e., the bond line thickness (BLT)), $∆R_{th}$ measured thermal resistance, contact area, and slope, respectively. According to Equations (3) and (4), the observed thermal resistance of the Al_2_O_3_–siloxane composite pads linearly depends on the thickness of the composite pads [11,12].

The viscosity of the Al_2_O_3_–siloxane composite paste was measured using a Brookfield viscometer (TVB-10, Toki Sangyo Co., Ltd., Shinbashi, Japan) at 25 °C. The rheological characteristics of the Al_2_O_3_–siloxane composite pastes were compared by evaluating the viscosity values obtained at low shear rates (0.025 to 0.20 s^−1^) to control the observed instability at high shear rates. This approach was used to investigate the effects of the high content ratio and surface treatment of Al_2_O_3_.

The hardness of the Al_2_O_3_–composite thermal pad was investigated as a mechanical performance indicator, which was used to characterize the degree of softness and hardness under specific conditions. Furthermore, the degree of hardness refers to the material’s resistance to local deformation, especially plastic deformation, and its indentation ability. The rubber hardness was evaluated using the ASTM D2240 method with a durometer. The hardness of the fabricated Al_2_O_3_–composite thermal pad was then measured using Shore 00–type indentor hardness. 

Finally, a thermogravimetric (TG) loss curve was obtained using a TG/differential thermal analyzer (STA 409PC, NETZSCH, Selb, Germany) for the thermogravimetric analysis of the silane surface treatment. This analysis was conducted under the conditions of 1000 °C in an air atmosphere at a heating rate of 274.15 K/min. TGA was performed under a minimum ambient air flow of 40 mL/min in a temperature range of 25 to 1000 °C.

## 3. Results and Discussion

### 3.1. Synthesis of the Al_2_O_3_ Nanoparticle Powder

**Figure 4:**
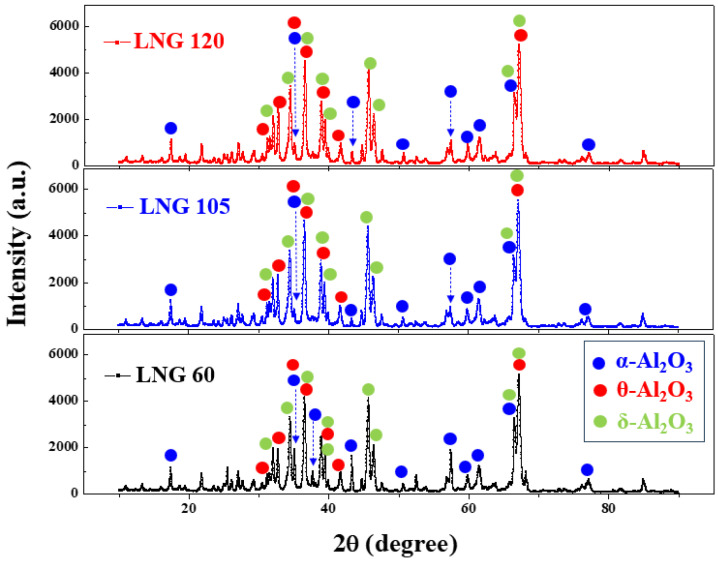
XRD patterns of the synthesized Al_2_O_3_ nanoparticle powders obtained using three different LNG flow rates.

Figure 4 shows the XRD patterns of the synthesized Al_2_O_3_ powders with three different LNG flow rates. The experimental results indicate that all Al_2_O_3_ powder samples have three crystal phases corresponding to δ–Al_2_O_3_ (PDF#04–021–8097), θ–Al_2_O_3_ (PDF#01–086–1410), and α–Al_2_O_3_ (PDF#01–071–1123). The quantified crystal phase is analyzed using the Rietveld method in combination with the obtained XRD spectra. The increase in the proportion of the δ phase from 85% to 88% when the LNG flow rates changed from 60 to 120 Nm^3^/h can be associated with a change in the decrease in the proportion of the α phase from 7% to 2%. In general, the Al_2_O_3_ powder has a γ–Al_2_O_3_ phase when obtained below 700 to 800 °C [13]. However, the Al_2_O_3_ powder obtained in this study at approximately 3000 °C above 800 °C has a complex crystalline phase of δ–, θ–, and α–Al_2_O_3_ [13]. The detailed Rietveld analysis results are summarized in Table 2.

**Figure 5:**
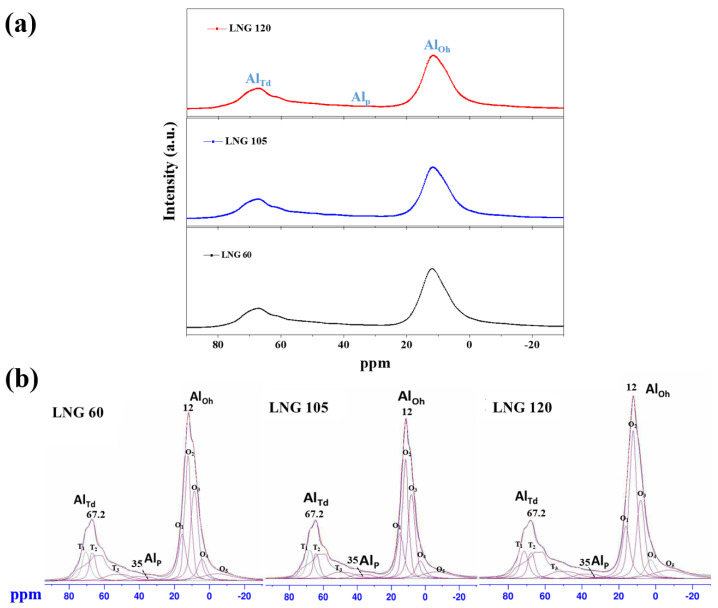
(**a**) ^27^Al solid NMR spectra and (**b**) their simulated peaks obtained using quadrupole coupling constants of the synthesized Al_2_O_3_ powders with three different LNG flow rates.

Figure 5 shows the high–resolution ^27^Al solid NMR spectra of the synthesized Al_2_O_3_ powders obtained with respect to three different LNG flow rates. All spectra consist of three major peaks centered at 12, 35, and 67.2 ppm, which can be attributed to the Al^3+^ cations in the octa (Al_Oh_), penta (Al_P_), and tetrahedral (Al_Td_) coordinations, respectively [14,15]. The surface of the Al_2_O_3_ crystal is regarded as a truncated plane of crystal consisting of coordinately unsaturated anion and cation sites. When the O–Al–O ratio of the basic Al_2_O_3_ structure increases, the Al_2_O_3_ structure is changed from a tetrahedral to an octahedral structure. In a stable octahedral structure, Al^3+^ cations have a strong ionic chemical bond and do not easily react with other ions. Meanwhile, Al^3+^ cations exhibit weak charge-balancing ionic interactions in the tetrahedral structure and easily react with other ions.

Because this reaction occurs easily within the tetrahedral structure, the viscosity of the Al_2_O_3_–siloxane composite paste may increase due to the resistance resulting from the free random movement and shear forces of the Al_2_O_3_ particles [15]. The ^27^Al NMR analysis results indicate that the characteristics of the tetra–, penta–, and octa–coordinated Al^+^ sites can affect the surface properties of Al_2_O_3_. The crystal phase of the Al_2_O_3_ powder was dominant, as confirmed by the δ–Al_2_O_3_ phases of the octahedral and tetrahedral coordinations. The quantified octahedral and tetrahedral coordinations of the Al^+^ site in the Al_2_O_3_ powder were compared to the sum of the individual fitting lines. The ratios of the Al^+^ sites (Al_Oh_/Al_Td_) in the three samples obtained when the LNG flow rate was varied from 60, 105, and 120 Nm^3^/h are listed in Table 3. The detailed peak ratios of the octahedral and tetrahedral sites are summarized in Table 3, which were calculated from the ^27^Al solid NMR spectra of the Al_2_O_3_ powders obtained under the three different LNG flow rates. 

The ^27^Al NMR results indicate that at an LNG flow rate of 120 Nm^3^/h, the ratio of Al^+^ sites (Al_Oh_/Al_Td_) slightly increased from 3.0% to 3.2% because of an increase in the ratio of octahedrons compared to that of tetrahedrons. Compared to the XRD results, the proportion of the δ–Al_2_O_3_ phase increased in the order of 85%, 87%, and 88% for LNG flow rates of 60, 105, and 120 Nm^3^/h, respectively. For the LNG flow rate of 105 Nm^3^/h, the synthesized Al_2_O_3_ powder showed a low peak ratio (Al_Oh_/Al_Td_) of the Al^+^ site. The comparatively more unstable tetrahedral structure became dominant on the surface of the systems, with a lower ratio of Al^+^ sites (Al_Oh_/Al_Td_). However, this is affected by the surface lattice distortions caused by molecular bonds such as O–Al–O and M–Al–O, owing to the inclusion of anionic impurities (M^+^) [16]. At an LNG flow rate of 120 Nm^3^/h, the Al_2_O_3_ powder has a high coordination of the octahedral phase with a high peak ratio (Al_Oh_/Al_Td_) of the Al^+^ site. The high coordination of the octahedral phase of the Al_2_O_3_ surface is more favorable toward the Al_2_O_3_ composite paste process for decreasing the resistance of the powder particle movement. This can help decrease the shear stress and viscosity, thereby increasing the powder packing density [16]. Therefore, we have established the optimum conditions for the LNG flow rate for spheriodization using frame fusion based on the ^27^Al NMR results.

**Figure 6:**
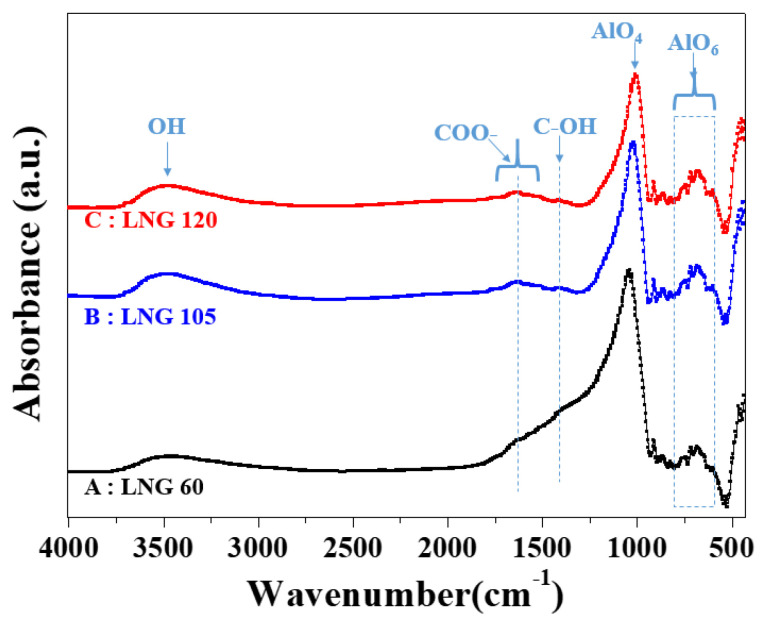
FTIR spectra of the synthesized Al_2_O_3_ powders with three different LNG flow rates.

Figure 6 shows the FTIR spectra of the synthesized Al_2_O_3_ powders with three different LNG gas flow rates. Based on the experimental results of all samples, the strong absorption peaks of Al_2_O_3_ at 3430, 1637, and 1413 cm^−1^ are the C–OH stretching vibration peak, –C=O stretching, and C–OH bending vibration peaks, respectively, thereby indicating the presence of oxygen–containing groups on carbonate [17]. These peaks are attributed to the residual carbonate formed via methane combustion due to incomplete flame fusion combustion. The peaks of the residual carbonate functional groups are reduced when the LNG flow rate is increased from 105 to 120 Nm^3^/h. 

### 3.2. Surface Treatment of the Al_2_O_3_ Powder

**Figure 7:**
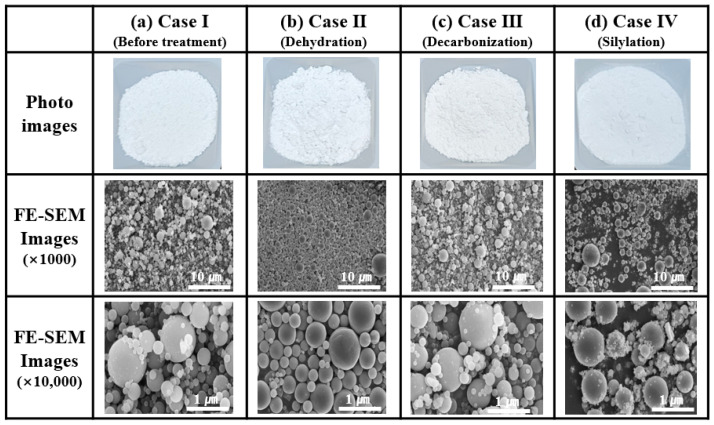
Photographs and FE-SEM images of surface–treated Al_2_O_3_ powders with various treatment conditions (Cases (**a**) I, (**b**) II, (**c**) III, and (**d**) IV).

Surface treatments (Cases I, II, III, and IV) of the spherical Al_2_O_3_ powders were conducted to investigate their effect on the agglomeration of Al_2_O_3_ powders. Figure 7 shows photographs and FE-SEM images of the surface-treated Al_2_O_3_ powders with various treatment conditions (Cases I, II, III, and IV). All Al_2_O_3_ powders showed spherical morphologies according to the images obtained before and after surface treatment. In Cases II and III, the treated Al_2_O_3_ powders were well separated and dispersed without agglomeration after undergoing surface treatment. The FE–SEM image results show that in Case IV, the nanoparticles dispersed more effectively compared to those in the other treatment conditions. Furthermore, Case II has a disadvantage in that suspended nanoparticles (100 nm or less) can be removed by a solvent during the dehydration-washing process, as shown in Figure 7b. Case IV suggests that the treated Al_2_O_3_ powders show a more densified nanoparticle separation distribution when compared to Cases II and III after undergoing surface treatment.

Table 4 lists the element compositions analyzed using ICP–OES quantification of the treated Al_2_O_3_ powders to investigate the effects of decreasing cation compositions (Si, Fe, Ca, Mg, and Na) resulting from Case II and Case III on electrical conductivity. The samples were prepared by mixing Al_2_O_3_ powder and DI in a ratio of 1:10. The supernatant was prepared for extraction after sonication for 10 min. In Case III, the Si element concentration decreased from 0.16 to 0.1 mg/L, and Fe, Ca, and Mg were detected at levels below 0.01 mg/L. In Case II, the Na element decreased from 5.02 to 0.74 mg/L. The total amount of major cations (Si, Fe, Ca, Mg, and Na) that affect electrical conductivity in spherical Al_2_O_3_ powders could be reduced to less than 1 ppm by combining Cases II and III. Moreover, when fabricating Al_2_O_3_–siloxane composite thermal pads, these impurities easily bind to compounds with non–bonding electron pairs on the Pt catalyst through coordinate covalent bonds. Therefore, a low concentration of trace impurities can improve the crosslinking of siloxane polymerization in the presence of a Pt catalyst [18]. 

### 3.3. Fabrication of Al_2_O_3_–Siloxane Composite Thermal Pads

**Figure 8:**
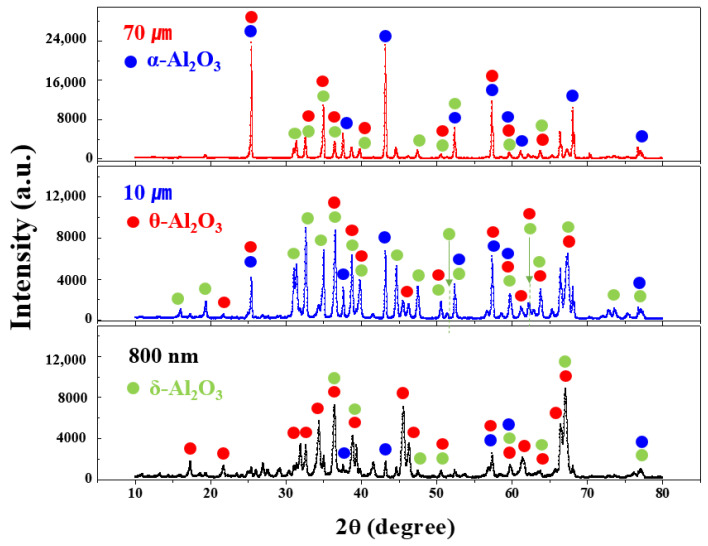
X-ray diffraction (XRD) patterns of the crystalline phase of the spherical Al_2_O_3_ powder with trimodal size distributions of 800 nm, 10 µm, and 70 µm.

Figure 8 shows the XRD patterns of the crystalline phase of the spherical Al_2_O_3_ powders with coarse (70 µm), fine (10 µm), and ultrafine (800 nm) trimodal size distributions. The XRD quantitative analysis results of the spherical Al_2_O_3_ powders applied in this test are shown in Figure 8. For the Al_2_O_3_ powder, the main phase is the δ-Al_2_O_3_ phase (91%), accompanied by α-Al_2_O_3_ (7%) and θ–Al_2_O_3_ (2%). For a particle size of 10 μm, the primary phase is θ– Al_2_O_3_ (62%), whereas for a particle size of 70 μm, the main phase is α–Al_2_O_3_ (64%). The detailed quantitative phase results are presented in Table 5. The crystal structure was quantified via Rietveld refinement analysis, and the peak shapes were fitted using a split pseudo–Voigt method.

**Figure 9:**
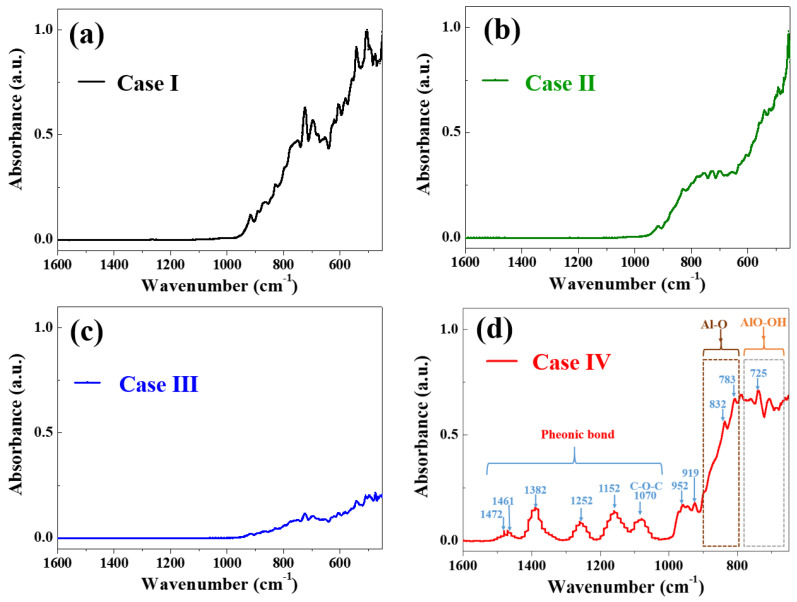
FTIR spectra of the treated Al_2_O_3_ powder with various surface treatments (Cases (**a**) I, (**b**) II, (**c**) III, and (**d**) IV).

Figure 9 shows the FT–IR spectra of the treated Al_2_O_3_ powder with various surface treatments (Cases I, II, III, and IV). In Case I, the surface of the untreated Al_2_O_3_ powder exhibited peaks at 840 to 784 cm^−1^ and 735 to 725 cm^−1^, which can be attributed to the stretching vibrations of Al–O in AlO_4_ and bending vibrations of AlO–OH, respectively [19,20]. In Case II, the OH peak of the treated Al_2_O_3_ powder decreased compared to that in Case I. For Case III, the OH peak of the treated Al_2_O_3_ powder decreased because of the heat treatment conducted at 750 °C for 1 h. After the silane treatment (Case IV), the surface of the treated Al_2_O_3_ powder had aromatic functional groups (1472 and 1461 cm^−1^), which corresponds to the C=C benzene ring vibrations [21]. It was confirmed that the aryl functional groups of silane were well attached to the surface of the Al_2_O_3_ powder.

**Figure 10:**
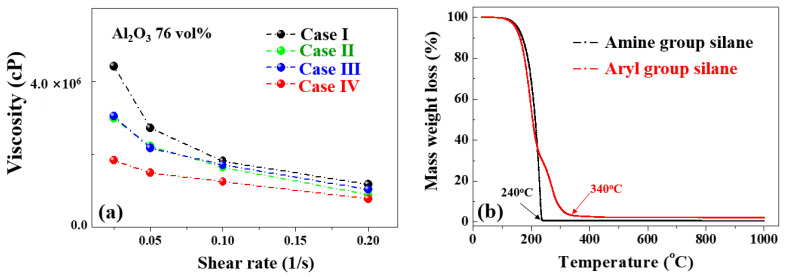
(**a**) Viscosities of Al_2_O_3_–siloxane composite pastes based on the shear rate with various surface treatments (Cases I, II, III, and IV) and (**b**) TGA results of silane applied to Al_2_O_3_–siloxane composite pastes (aryl silane for Case IV, amine silane (APTES) for reference).

Figure 10a shows the viscosities of the Al_2_O_3_–siloxane composite pastes with an Al_2_O_3_ concentration of 76 vol% according to the shear rate with various surface treatments (Cases I, II, III, and IV) measured using a viscometer at 25 °C. For Cases II and III, the viscosity of the Al_2_O_3_–siloxane composite pastes decreased due to the removal of the carbonate and hydroxyl groups in the Al_2_O_3_–siloxane composite paste compared to Case I. 

When the absorbed hydroxyl of Al_2_O_3_ is removed, bonding with hydrophobic siloxanes can be promoted, and viscosity can be reduced in the Al_2_O_3_–siloxane pastes [22,23].

For Case IV, the viscosity of the Al_2_O_3_–siloxane composite paste was decreased at the fixed volume ratio of the Al_2_O_3_ powder when compared to Case I. 

Figure 10b shows the TGA thermogram results of silane obtained using the treated Al_2_O_3_ powder (Case IV). The thermal weight loss of aryl silane and amine silane (3–aminopropyl-triethoxysilane, molecular weight of 221.37 g/mol) occurred at ~240 and 340 °C, respectively. The total weight losses of the aryl silane and amine silane were 2.07% and 0.44% at 1000 °C, respectively. The weight loss of aryl silane was observed in the range of 250 to 450 °C, which may have resulted from the removal of the double bond (sp^2^) in carbon. The gradient of the weight loss in this range can be attributed to the different oxidation reaction rates of carbon species (sp^2^ and sp^3^) [24]. The double bond (sp^2^) of aryl silane can contribute to more stable thermal decomposition and improve the thermal resistance compared to that of amine silane [23].

**Figure 11:**
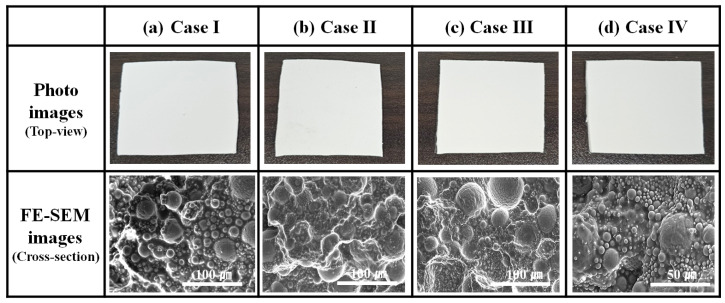
Photographs and FE–SEM cross–section images of Al_2_O_3_–siloxane composite pads fabricated using composite pastes with various surface treatments (Cases (**a**) I, (**b**) II, (**c**) III, and (**d**) IV).

Figure 11 shows photographs and FE-SEM cross-section images of Al_2_O_3_–siloxane composite pads with 76 vol% prepared by using Al_2_O_3_–siloxane composite pastes with various surface treatments (Cases I, II, III, and IV). Herein, to make the cross–sectional sample of thermal pads for the FE–SEM measurement, the thermal pad sample was prepared using physical fixation methods, such as immersion freezing and cryofixation. The Al_2_O_3_–siloxane composite thermal pad was plunged into liquid nitrogen (LN_2_) and allowed to drop to −175 °C. In the cryofixation step, the pad was cut quickly to minimize cross–sectional damage to the sample fracture. After reaching room temperature, the cooled cross–sectional specimen was sufficiently dried in the oven at 100 °C for 24 h.

In all cases, the Al_2_O_3_ powder with a trimodal particle size was distributed in the siloxane matrix. In Case I, the cross-sectional surface of the Al_2_O_3_–siloxane composite thermal pads was relatively rough. The edges of the bare Al_2_O_3_ were partially exposed, and some regions of the Al_2_O_3_ particle were not covered by the siloxane. This can be attributed to the curing failure of the OH groups of siloxanes onto Al_2_O_3_ caused by the residual moisture prior to treatment [25]. The OH group from water may inhibit the catalysis of platinum (Pt) upon its addition to silicon. The OH group reacts with the unreacted vinyl (Vi) side–groups of the Si–O backbone that is cross–linked with the Pt catalyst via polymerization [18]. In Case IV, the silylated Al_2_O_3_ powder formed flexible bonds with the siloxane matrix. Al_2_O_3_ nanoparticles have a large specific surface area (adsorbent BET area of 2.47 m^2^/g at 800 nm, which is higher than that of 0.1 m^2^/g for 10–70 µm), and these particles were well distributed around the larger micrometer–sized particles, as shown in the FE-SEM cross–section images of the Al_2_O_3_–siloxane composite thermal pads. 

As a generalized theory of heat conduction, Loeb’s model (also referred to as Sankar−Loeb’s model) is mainly used to understand the thermal energy transport in a polymer matrix. According to Loeb’s model, the thermal conductivity of the composite thermal pad depends on the size distribution and orientation of the solid Al_2_O_3_ [26]. 

In this study, we attempted to improve the thermal conductivity of Al_2_O_3_-siloxane composite thermal pads by improving the compatibility and adhesion between Al_2_O_3_ particles and the siloxane matrix using various surface treatments (Cases I, II, III, and IV). 

In Case IV, the strong interactions caused by aryl silylation on the surface of the Al_2_O_3_ nanoparticles between the cross-linked Si–Vi and H–Si systems improved the bonding strength, thereby resulting in a higher mechanical strength. Considering the silylation mechanism, the silylated Al_2_O_3_ powder first reacts with the siloxane matrix. 

The hydroxyl functional groups of the Al_2_O_3_ powder react with aryl silane through hydrolysis, which decreases the surface tension. The silane–treated Al_2_O_3_ becomes bonded to the non–polar siloxane, resulting in aryl groups in the polymeric network. 

Finally, a reaction occurs in parallel between the siloxane vinyl monomers and the silicone hydride crosslinker via Pt-catalyzed hydrosilylation. The Al_2_O_3_–siloxane composite network includes physically and chemically linked species, improving the compatibility between Al_2_O_3_ and the siloxane resin matrix. The thermal energy transport in this system improved its mechanical and thermal conductivity properties. Furthermore, this fully connected network contributed to reducing the viscosity of the paste [27]. 

The FE-SEM results confirmed the densified distribution of the Al_2_O_3_ nanoparticles, and the siloxane coverage of the Al_2_O_3_ particles was improved after surface treatment.

**Figure 12:**
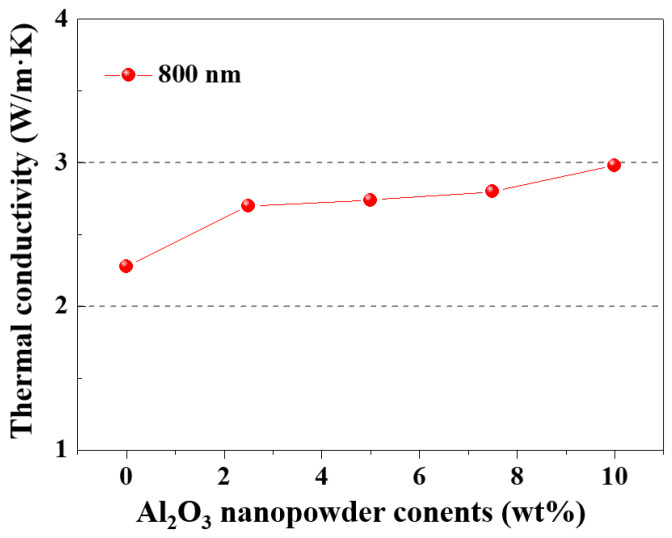
Thermal conductivity of Al_2_O_3_–siloxane composite thermal pads with increasing the amount of nanoparticle powder for Case I.

Figure 12 shows the thermal conductivity of Al_2_O_3_–siloxane composite thermal pads prepared using an Al_2_O_3_ nanoparticle powder (800 nm) when the amount of Al_2_O_3_ nanoparticle powder is changed from 0 wt% to 10 wt% for Case I. In this experiment, the mass ratio of 70 and 10 μm micro–sized Al_2_O_3_ powder was tested by fixing it under total powder conditions of 76 vol% at a ratio of 6:4, respectively. The thermal conductivity improved noticeably as the Al_2_O_3_ nanoparticle powder content of the composite pad increased from 0 wt% to 10 wt%. Specifically, the thermal conductivity of the composite pads increased by more than 30% compared to the Al_2_O_3_–siloxane composite thermal pads without nanoparticles. This suggests that the addition of Al_2_O_3_ nanoparticle powder enhances the thermal conductivity of the composite material, potentially due to improved thermal pathways created by the nanoparticles within the matrix. In particular, this improvement in thermal conductivity properties is effective due to the Farris effect under conditions of high Al_2_O_3_ filler content of 50 vol% or more [10].

**Figure 13:**
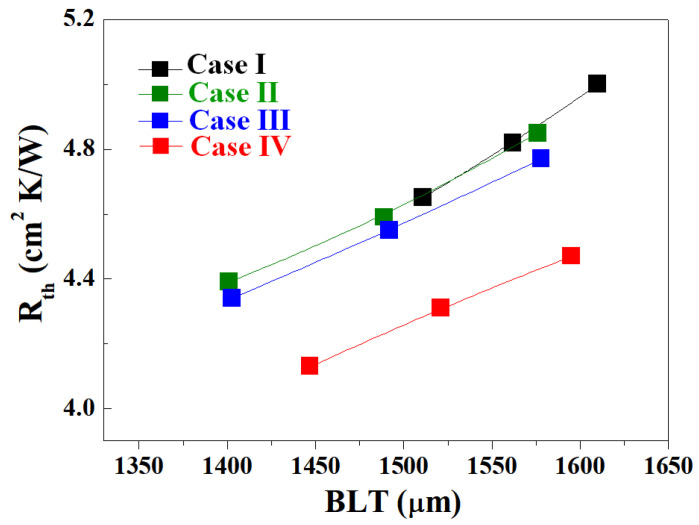
Thermal resistance (R_th_) of Al_2_O_3_–siloxane composite thermal pads with various sample thicknesses (BLTs).

Figure 13 shows the thermal resistance (R_th_) of Al_2_O_3_–siloxane composite thermal pads with various sample thicknesses (BLTs) obtained using Al_2_O_3_ powder samples with various surface treatments. The R_th_ values of the Al_2_O_3_-siloxane composite thermal pads were measured at approximately 275.6 to 344.5 kPa. As the Al_2_O_3_ content increased, the thermal resistances of the composites in Case I (76 vol%) and Cases II, III, and IV (79 vol%) decreased rapidly. In the Al_2_O_3_ powder samples for Cases II, III, and IV, the slope of R_th_ and BLT decreased to a greater extent than that of the sample before treatment (Case I). In Case IV, the R_th_ of the Al_2_O_3_–siloxane composite thermal pad (4.20 cm^2^·K/W at 1510 µm) decreased by almost 10% in comparison to that in Case I (4.67 cm^2^·K/W at 1510 µm). For Cases II and III, the R_th_ values of the Al_2_O_3_–siloxane composite thermal pads were 4.38 cm^2^·K/W at 1400 µm and 4.32 cm^2^·K/W at 1398 µm, respectively. These significantly decreased R_th_ values could be attributed to the high thermal conductivity and low BLT of the Al_2_O_3_–siloxane composite thermal pads achieved using an appropriate pressure.

**Figure 14:**
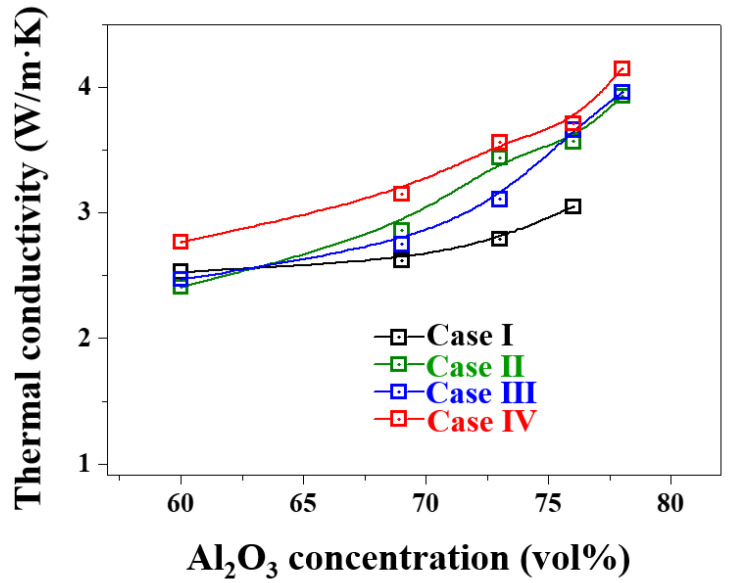
Thermal conductivity of Al_2_O_3_–siloxane composite thermal pads before and after surface treatments with different concentrations of Al_2_O_3_ powder.

Figure 14 shows the thermal conductivity of the prepared Al_2_O_3_–siloxane composite thermal pads with various volume fractions of Al_2_O_3_ powder from 60 to 78 vol%. 

The experimental results indicated that the thermal conductivity of the Al_2_O_3_–siloxane composite thermal pads increased with an increase in the volume fraction of the Al_2_O_3_ filler in the siloxane matrix. At 78 vol% Al_2_O_3_ (4.15 W/m·K) in Case IV, the aryl silylation Al_2_O_3_ powder enhanced the heat transfer performance of the Al_2_O_3_–siloxane composite thermal pads. The thermal conductivity of the silylated Al_2_O_3_–siloxane composite thermal pads was considerably higher than that of the untreated Al_2_O_3_-siloxane composite thermal pads (3.05 W/m·K). For Cases II and III, the thermal conductivities of the 78 vol% Al_2_O_3_-siloxane composite were 3.93 and 3.96 W/m·K, respectively. The thermal conductivity of the Al_2_O_3_–siloxane composite was enhanced by 22% when compared to that of Case I. In Case IV, the thermal conductivity of the Al_2_O_3_–siloxane composite thermal pads improved by 26.5% at a high filler content compared to Case I. This improvement in thermal conductivity can be attributed to the thermal bridge effect at the interface between Al_2_O_3_ and siloxane. These surface treatments (Cases II, III, and IV) resulted in the formation of more interconnected thermal pathways and contributed to the enhancement of adhesive contact as the LNG flow rate increased via the relaxation of the internal friction between Al_2_O_3_ and siloxane [28]. The cross–sections of the Al_2_O_3_–siloxane composite thermal pads were improved considering the distribution of the Al_2_O_3_ particles and siloxane coverage, as shown in Figure 11.

Table 6 presents the thermomechanical and electrical properties of the Al_2_O_3_–siloxane composite thermal pads with various process parameters, such as volume fraction of Al_2_O_3_ nanoparticle powder, thickness of composite pads, and surface–treated Al_2_O_3_ nanoparticle powder (Cases II, III, and IV). The hardness of the Al_2_O_3_–siloxane composite pads was evaluated under various specific pressure conditions. According to the commercialized analysis standard reference, the thermal conductivity of the Al_2_O_3_–siloxane composite thermal pads had a range of 0.2 to 1.9 W/m·K, meaning that they showed a hardness range of 80 to 90 using Shore 00-type indentor hardness [29]. 

The thermal conductivities of the Al_2_O_3_–siloxane composite pads were then calculated based on the variation in the thermal resistance at a pressure of 344.5 kPa and a temperature of 50 °C. For Case I, the thermal conductivity of the Al_2_O_3_–siloxane composite thermal pads was from 2.53 to 3.05 W/m·K, and the hardness ranged from 46H to 69H. In Case IV, the Al_2_O_3_ particles were treated with aryl silane before preparing the composite to enhance the interfacial contact between Al_2_O_3_ and the matrix. The thermal conductivity pads exhibited a thermal conductivity of 2.77 and a maximum value of 4.15 W/m·K at harnesses ranging from 57 HS to 85 HS. The Al_2_O_3_–siloxane composite thermal pads with high hardness values at high pressure exhibited a high compression rate, a short thermal conductivity path, a short heat transfer time, and better thermal conductivity compared to the low–hardness Al_2_O_3_–siloxane composite thermal pads. The Al_2_O_3_–siloxane composite thermal pads showed resistance to deformation when they exhibited high hardness. This value suggested that the hardness of the pads contributed to their structural integrity and ability to maintain their shape under applied pressure or load. High hardness often correlates with increased stiffness and resistance to compression, which are desirable properties for thermal interface materials like composite pads, as they need to maintain consistent thermal contact between surfaces without undergoing significant deformation. Further, the thermal conductivity of the Al_2_O_3_–siloxane composite pads was enhanced when the hardness was increased in the surface–treated Al_2_O_3_ nanoparticle powders (Cases II, III, and IV) compared to Case I.

**Figure 15:**
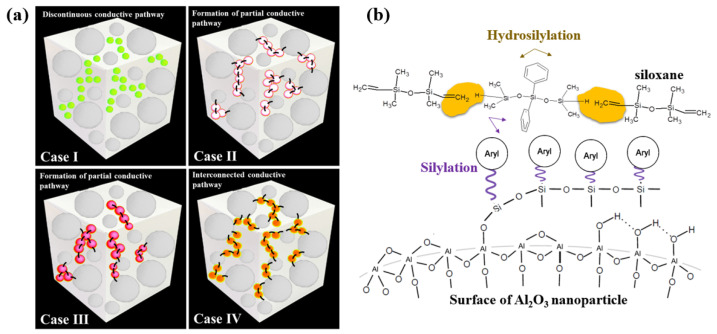
(**a**) Schematic diagram of the heat-interconnected pathway in the Al_2_O_3_-siloxane composite thermal pads prepared by using various surface–treated Al_2_O_3_ nanoparticle powders (Cases I, II, III, and IV). (**b**) The chemical interaction of Al_2_O_3_-siloxane composite pads during both aryl functional silylation and hydrosilylation cross–linking reactions with a Pt–based catalyst.

Figure 15a shows the schematic diagram of the heat-interconnected pathway in the Al_2_O_3_–siloxane composite thermal pads prepared by using various surface–treated Al_2_O_3_ nanoparticle powders (Cases I, II, III, and IV). In all cases, the Al_2_O_3_ nanoparticle with a trimodal particle size was distributed in the siloxane polymer matrix. In Case I, the Al_2_O_3_ was partially exposed, and some regions of the Al_2_O_3_ particle did not cover the siloxane. This can be attributed to the curing failure of the OH groups of siloxanes onto Al_2_O_3_ caused by the residual moisture. The OH group from water may inhibit the catalysis of Pt upon its addition to silicon. The OH group reacts with the unreacted vinyl (Vi) side–groups of the cross–linked Si–O backbone by Pt catalyst polymerization [23,25]. In Case IV, the silylated Al_2_O_3_ nanoparticle formed flexible bonds with the siloxane matrix. Al_2_O_3_ nanoparticle powder has a large specific surface area, and these particles were well distributed around the larger micrometer–sized particles, as shown in the FE–SEM cross-section results (Figure 11d). Therefore, the silylated Al_2_O_3_ nanoparticles make it easy to form the thermal conductivity channel network, which is more stable through a dense stacking structure [1,10,18,29]. For this reason, the thermal conductivity of the composite thermal pad applied with the silylated Al_2_O_3_ nanoparticles was improved.

To understand the mechanism of the silylated Al_2_O_3_ nanoparticles for thermal conductivity, Figure 15b shows the chemical interaction of Al_2_O_3_–siloxane composite pads during both aryl functional silylation and hydrosilyation cross–linking reactions with a Pt-based catalyst. In Case IV, the Al_2_O_3_ particles showed a figure of the relations between structure and property performance with aryl silane before preparing the composite to enhance the interface contact between Al_2_O_3_ and the siloxane matrix. The silylated Al_2_O_3_ nanoparticle first reacts with the siloxane polymer matrix, and then the hydroxyl functional groups of the Al_2_O_3_ particle react with aryl silane through hydrolysis for surface functionality switching from hydrophilicity to hydrophobicity, which decreases the surface tension. The silylated Al_2_O_3_ nanoparticles become bonded to the non-polar siloxane, resulting in aryl groups in the polymeric network. This reaction occurs in parallel between the siloxane vinyl monomers and the silicone hydride crosslinker via Pt–catalyzed hydrosilylation. The Al_2_O_3_–siloxane composite network includes physically and chemically linked species, improving the compatibility between Al_2_O_3_ and the siloxane polymer matrix. The thermal energy transport in this system improved its mechanical and thermal conductivity properties. In addition, these reactions in the fully connected network contributed to reducing the viscosity of the paste [26].

## 4. Conclusions

This study investigated the structural properties of spherical Al_2_O_3_ powders prepared by flame fusion with three different LNG flow rates. When analyzing the Al_2_O_3_–siloxane composite thermal pads, the spherical Al_2_O_3_ powders were subjected to various surface treatments, such as before treatment (Case I), dehydration (Case II), decarbonization (Case III), and silylation (Case IV). In addition, the spherical Al_2_O_3_ powders and Al_2_O_3_-siloxane composite thermal pads were characterized using FE–SEM, XRD, FT–IR, NMR, ICP–OES, and thermal conductivity measurements. 

In Cases II and III, the treated Al_2_O_3_ powders exhibited reduced carbonate and hydroxyl molecule components. The synthesized spherical Al_2_O_3_ nanoparticle powder was mainly composed of the δ–Al_2_O_3_ phase (88%) and exhibited a stable surface with a high octahedral ratio (A_Oh_/A_Td_) of 3.2%, which was confirmed by XRD and NMR analyses. 

Moreover, Al_2_O_3_-siloxane composite thermal pads were fabricated using surface–treated spherical Al_2_O_3_ powders with trimodal size distributions, which resulted in high thermal conductivity. The Al_2_O_3_–siloxane composite thermal pads exhibited high hardness and excellent heat transfer at higher amounts of Al_2_O_3_ powder. For Case IV, the thermal conductivity of the Al_2_O_3_–siloxane composite thermal pads was enhanced by 26.5% compared to that of Case I. In this study, the spheriodized Al_2_O_3_ nanoparticles exhibiting an 88% δ–phase were optimally synthesized using the flame fusion process to achieve a trimodal–sized paste formulation. The surface functionalization of these systems improved their high thermal conductivity, indicating their potential for use as high-thermal–conductivity TCMs.

## Figures and Tables

**Table 1:** Mixing ratio of the synthesized spherical Al_2_O_3_ powder for Al_2_O_3_–siloxane composite thermal pads.

The Synthesized Spherical Al_2_O_3_ Powder	Particle Size of the Al_2_O_3_ Powder		
	70 μm	10 μm	800 nm
Mixing ratio (wt. %)	55	36	9

**Table 2:** Quantitative phase analysis results of Al_2_O_3_ nanoparticle powders with three different LNG flow rates.

Crystal Phase (%)	LNG Flow Rates (Nm^3^/h)		
	60 Nm^3^/h	105 Nm^3^/h	120 Nm^3^/h
δ	85	87	88
θ	8	10	10
α	7	3	2

**Table 3:** Detailed octahedral and tetrahedral sites calculated from the ^27^Al solid NMR spectra for Al_2_O_3_ powders with three different LNG flow rates.

LNG Flow Rates (Nm^3^/h)	Al_Td_ (%)	Al_P_ (%)	Al_Oh_ (%)	Al_Oh_/Al_Td_
60 Nm^3^/h	33	2.6	100	3.0
105 Nm^3^/h	34	2.9	100	2.9
120 Nm^3^/h	31	2.7	100	3.2

**Table 4:** Elemental compositions obtained by ICP–OES quantification for the treated Al_2_O_3_ powders with the various treatment conditions of (a) Cases I, (b) II, and (c) III.

Case Study	Element Compositions (mg/L)				
	Si	Fe	Ca	Mg	Na
Case I (before treatment)	0.16	<0.01	0.51	0.08	5.02
Case II	0.12	<0.01	0.65	0.04	0.74
Case III	0.11	<0.01	<0.01	<0.01	4.46

**Table 5:** Relative XRD quantification results of the spherical Al_2_O_3_ powder with trimodal size distributions for Al_2_O_3_–siloxane composite thermal pads.

Crystal Phase (%)	Particle Size of Al_2_O_3_ Powder		
	800 nm	10 µm	70 µm
δ	91	19	1
θ	2	62	35
α	7	19	64

**Table 6:** Thermal conductivity and mechanical hardness properties of Al_2_O_3_–siloxane composite thermal pads with various process parameters, such as volume fraction of Al_2_O_3_ nanoparticle powder, thickness of composite pads, and surface-treated Al_2_O_3_ nanoparticle powder (Cases II, III, and IV).

Case Studies	Volume Fraction (%)	Thickness (mm)	Hardness (HS)	Thermal Conductivity (W/m·K)
Case I	60	1.92	46	2.53
	69	2.40	55	2.62
	73	2.40	67	2.79
	76	2.43	69	3.05
Case II	60	2.20	59	2.41
	69	2.28	57	2.86
	73	2.21	72	3.44
	76	2.35	77	3.57
	79	2.38	80	3.93
Case III	60	2.52	53	2.47
	69	3.09	51	2.75
	73	3.02	71	3.11
	76	3.01	79	3.66
	79	3.13	80	3.96
Case IV	60	2.52	57	2.77
	69	2.92	72	3.15
	73	3.05	77	3.56
	76	2.90	79	3.71
	79	3.02	85	4.15

## Data Availability

Data are contained within the article.

## References

[1] Xu Z., Zhang C., Li Y., Zou J., Li Y., Yang B., Hu R., Qian Q. (2023). Effect of the alumina micro-particle sizes on the thermal conductivity and dynamic mechanical property of epoxy resin. PLoS ONE.

[2] Klimov A., Bakeev I., Zenin A. (2023). Electron-beam processing of aluminum-containing ceramics in the forevacuum pressure range. Ceramics.

[3] Dong X., An Q., Zhang S., Yu H., Wang M. (2023). Porous ceramics based on high-thermal-stability Al_2_O_3_–ZrO_2_ nanofibers for thermal insulation and sound absorption applications. Ceram. Int..

[4] He S., Li Y., Zong X., Wu S. (2023). The effect of AlN content on the properties of Al_2_O_3_-AlN composite ceramics fabricated by digital light processing. Crystals.

[5] Xu X., Chen J., Zhou J., Li B. (2018). Thermal conductivity of polymers and their nanocomposites. Adv. Mater..

[6] Zhang H., Zhang J. (2022). Rheological behaviors of plasticized polyvinyl chloride thermally conductive composites with oriented flaky fillers: A case study on graphite and mica. J. Appl. Polym. Sci..

[7] Varenik M., Nadiv R., Levy I., Vasilyev G., Regev O. (2017). Breaking through the solid/liquid processability barrier: Thermal conductivity and rheology in hybrid graphene–graphite polymer composites. ACS Appl. Mater. Interfaces.

[8] Wen H.L., Chen Y.Y., Yen F.S., Huang C.Y. (1999). Size characterization of θ-and α-Al_2_O_3_ crystallites during phase transformation. Nanostruct. Mater..

[9] Eom S.H., Pee J.H., Lee J.K., Hwang K.T., Cho W.S., Kim K.J. (2009). Effects of Chemical composition and particle size of starting aluminum source on the spheroidization in the flame fusion process. J. Powder Mater..

[10] Barnes H.A., Hutton J.F., Walters K. (1993). An Introduction to Rheology.

[11] Culham J.R., Teertstra P., Savija I., Yovanovich M.M. Design, Assembly and commissioning of a test apparatus for characterizing thermal interface materials. Proceedings of the ITherm 2002—Eighth Intersociety Conference on Thermal and Thermomechanical Phenomena in Electronic Systems.

[12] Kempers R., Kolodner P., Lyons A., Robinson A.J. (2009). A high-precision apparatus for the characterization of thermal interface materials. Rev. Sci. Instr..

[13] Kovarik L., Bowden M., Andersen A., Jaegers N.R., Washton N., Szanyi J. (2020). Quantification of high-temperature transition Al_2_O_3_ and their phase transformations. Angew. Chem. Int. Ed..

[14] O’Dell L.A., Savin S.L., Chadwick A.V., Smith M.E. (2007). A ^27^Al MAS NMR study of a sol–gel produced alumina: Identification of the NMR parameters of the θ-Al_2_O_3_ transition alumina phase. Solid State Nucl. Mag. Resonance.

[15] Xu S., Jaegers N.R., Hu W., Kwak J.H., Bao X., Sun J., Wang Y., Hu J.Z. (2021). High-field one-dimensional and two-dimensional ^27^Al magic-angle spinning nuclear magnetic resonance study of θ-, δ-, and γ-Al_2_O_3_ dominated aluminum oxides: Toward understanding the Al sites in γ-Al_2_O_3_. ACS Omega.

[16] Chen L., Liu K., Han P., Yang B., Feng L. (2020). Calculation and analysis of the structure and viscosity of B_2_O_3_-regulated CaO-Al_2_O_3_-based mold fluxes. J. Chem..

[17] Köck E.M., Kogler M., Bielz T., Klötzer B., Penner S. (2013). In situ FT-IR spectroscopic study of CO_2_ and CO adsorption on Y_2_O_3_, ZrO_2_, and yttria-stabilized ZrO_2_. J. Phys. Chem. C.

[18] Miller D.C., Kempe M.D., Muller M.T., Gray M.H., Araki K., Kurtz S.R. (2016). Durability of polymeric encapsulation materials in a PMMA/glass concentrator photovoltaic system. Prog. Photovolt. Res. Appl..

[19] Goldberg M.A., Protsenko P.V., Smirnov V.V., Antonova O.S., Smirnov S.V., Konovalov A.A., Vorckachev K.G., Kudryavtsev E.A., Barinov S.M., Komlev V.S. (2020). The enhancement of hydroxyapatite thermal stability by Al doping. J. Mater. Res. Technol..

[20] Sergeeva A.V., Zhitova E.S., Nuzhdaev A.A., Zolotarev A.A., Bocharov V.N., Ismagilova R.M. (2020). Infrared and Raman spectroscopy of ammoniovoltaite,(NH_4_)_2_Fe^2+^ 5Fe^3+ 3^Al (SO_4_)_12_(H_2_O)_18_. Minerals.

[21] Ahmad S., Farrukh M.A. (2012). Anti-microbial activities of sulfonamides using disc diffusion method. Park. J. Pharm. Sci..

[22] Atayde C., Doi I. (2010). Highly stable hydrophilic surfaces of PDMS thin layer obtained by UV radiation and oxygen plasma treatments. Physica Status Solidi C.

[23] Drake K., Mukherjee I., Mirza K., Ji H.F., Wei Y. (2011). Phenylethynyl and phenol end-capping studies of polybiphenyloxydiphenylsilanes for cross-linking and enhanced thermal stability. Macromolecules.

[24] Osswald S., Yushin G., Mochalin V., Kucheyev S.O., Gogotsi Y. (2006). Control of sp2/sp3 carbon ratio and surface chemistry of nanodiamond powders by selective oxidation in air. J. Am. Chem. Soc..

[25] Rossi A.M., Murphy T.E., Reipa V. (2008). Ultraviolet photoluminescence from 6H silicon carbide nanoparticles. Appl. Phys. Lett..

[26] Mohanachandran Nair Sindhu S., Sankaranarayana Iyer S. (2019). Generalized theory of thermal conductivity for different media: Solids to nanofluids. J. Phys. Chem. C.

[27] Ko E., Choi S.S. (2014). Characterization and formation of chemical bonds of silica-coupling agent-rubber. Elastomers Compos..

[28] Dou Z., Zhang B., Xu P., Fu Q., Wu K. (2023). Dry-contact thermal interface material with the desired bond line thickness and ultralow applied thermal resistance. ACS Appl. Mater. Interfaces.

[29] Li Y.-T., Liu W.-J., Shen F.-X., Zhang G.-D., Gong L.-X., Zhao L., Song P., Gao J.-F., Tang L.-C. (2022). Processing, thermal conductivity and flame retardant properties of silicone rubber filled with different geometries of thermally conductive fillers: A comparative study. Compos. Part B.

